# The dialectics of disaster: Considerations on hazards and vulnerability in the age of climate breakdown, with a brief case study of Khuzestan

**DOI:** 10.4102/jamba.v15i1.1588

**Published:** 2023-12-26

**Authors:** Andreas Malm

**Affiliations:** 1 Human Ecology Division, Lund University, Lund, Sweden

**Keywords:** dialectics, vulnerability, coastline, Marxism, climate breakdown

## Abstract

In a historical moment inundated by disasters, understanding and conceptualising the phenomenon is a matter of some importance. No framework for doing so has been more productive than that developed by Wisner and his colleagues. But their so-called ‘Progression of Vulnerability’ (pressure and release [PAR] model) framework was conceived before the onset of the climate crisis. And that crisis, as the saying goes, changes everything.

**Contribution:**

What follows is an immanent critique of the framework, with an eye towards shifting some of its parameters in order to account for the process of climate breakdown now multiplying disasters across the globe.

## Climate stability in the rise of critical vulnerability studies

The history of vulnerability studies will be familiar to many readers (e.g. Bankoff 2018), but some recapitulation is essential for bringing out the analytical problem at hand. In the post-war decades, Western research on natural hazards tended to assume that disaster was the result of an extreme geophysical occurrence: a flood, a storm, an earthquake. Vulnerability, it followed, was a function of the corresponding geophysical features into which the occurrence crashed: the inclination of a hillside, the flatness of a coastline, proximity to a major fault. It was in the *nature* of such collisions to produce harm. Causal arrows ran in straight lines from environment to population, humans stationed at the receiving end – or, as it were, crushed between two forces of nature (Eakin & Luers 2006:369; Hewitt 1983b:5–6; O’Brien et al. 2007:75–76). This paradigm has been variously designated ‘the risk-hazard model’, ‘the technocratic view’, ‘the biophysical discourse’ and ‘geophysicalism’; here, we shall stick with the latter term. Geophysicalism reigned supreme in Western academia until the 1970s, when it came under fire from young scholars, among them Wisner, who worked under the influence of Marxism. They wanted to take ‘the naturalness out of natural disasters’ (O’Keefe, Westgate & Wisner 1976).

In a sense, these iconoclasts simply returned the concept of vulnerability to its etymological roots. The Latin term *vulnerabilis* was ‘used by the Romans to describe the state of a soldier lying already wounded on the battlefield, i.e. already injured [and] therefore at risk from further attack’ (Kelly & Adger [2000] 2009:163). On this classical view, vulnerability is a condition inflicted on someone by some human antagonist. It makes the former liable to receive the next strike as a deathblow. ‘Disaster’ is the name for the fall of the blow; or, it ‘marks the interface between an extreme physical phenomenon and a vulnerable human population’, as Wisner and colleagues put it in their seminal early paper in *Nature* (O’Keefe et al. 1976:566). The 1970s also appeared to be a moment of disasters galore. Data indicated a surge in their number and casualty tolls. What accounted for this disturbing trend? In a move that cleaned the slate for their emerging framework, Wisner and colleagues argued:

No major geological or climatological changes over the last 50 years adequately explain the rise. There is little argument about geological change, but there has been much mystifying argument about climatic change, especially following the prolonged drought over the African and Asian continents. But no firm conclusion can be drawn about changing climatic conditions from available evidence. Randall Baker at the Development Studies School of the University of East Anglia recently reviewed all the evidence of climatic change in Africa and offered the Scottish judgment of ‘case not proven’. Even if some long-term change was observable it would not explain the increase in disaster occurrence observed in the data. (Wisner, O’Keefe & Westgate 1977)

The last statement is hard to make sense of as anything else than an axiomatic rejection. Had these words been written today, they would count as climate denial. But they were, of course, penned a decade before modern climate science matured, and therefore they escape that tarnish. The substantial point is that Wisner et al. erected their model on the premise of a stable climate system. Their deduction continued:

If it is accepted that there has been no major geological and climatological change in recent years, then it can be assumed that the probability of the extreme physical occurrence is constant.^1^ If the probability is constant, then logically the explanation of the increasing numbers of disasters must be sought in an explanation of the growing vulnerability of the population to extreme physical events. (O’Keefe et al. 1976; cf. Susman, O’Keefe & Wisner 1983:265)

In the inaugural issue of the journal *Disasters*, the case against climatic change was restated and the argument taken to its conclusion:

More and more people are becoming vulnerable to the occurrence of certain physical events which have been occurring with a certain mathematically reconstructable probability for centuries, if not millennia. It is in the phenomenon of vulnerability – that is, on the human side of the man-nature relationship – that an explanation is to be sought. (Wisner, O’Keefe & Westgate 1977:48)

Society, not nature, produces the death sentence for multitudes. Vulnerability, Wisner et al. contended, is a function of the unequal access to resources. Those with few resources – the poor, broadly speaking – are vulnerable to any given hazard, not because of the geophysical nature of the occurrence or its setting, but because of the way society is organised. If disasters become more common and destructive, it must – given the regularity of the hazards themselves: landslides, droughts, cyclones and the rest – be because of ever-deeper wounds sustained on the battlefield, in the form of impoverishment. Here Wisner et al. drew on the scholarship of underdevelopment so vibrant in the 1970s. ‘As resources continue to be controlled by a minority, the real standard of living drops for much of the population’ (O’Keefe et al. 1976:566). That is why people are left exposed to the full force of natural blows: they ‘die in disasters chiefly because insufficient money is spent saving lives’ (Anon 1977:1), the first editorial of the journal *Disasters* declared. As an alternative approach, this had much logic and data to speak for it.

Over the 1980s and 1990s, the counter-paradigm solidified under the labels ‘contextual’, ‘second generation’, ‘social’ and ‘critical’ vulnerability studies (cf. Bolin 2007); here, we shall use the latter term. In the landmark volume *Interpretations of Calamity: From the Viewpoint of Human Ecology*, contributors pressed home one cardinal idea: disasters are not chance events or ‘acts of God’ that erupt into ordinary life. Rather they should be seen as the starkest *truth* about that life, whose inner structure they bring to light (e.g. Hewitt 1983b:25).^2^ In his chapter, Michael Watts drew on research from northern Nigeria, later elaborated into a classic of political ecology: during a drought in the region, rich households stood the test thanks to the large size of their cattle herds and other assets, whereas the poorer ones bit the dust. Some owned the means for survival, others did not. Deaths and losses were not in any profound sense caused by the drought – it was at most ‘a catalyst’ – but rather by selective pressures inhering in the prevailing property relations (Watts 1983:258). In this scheme of things, what goes on in nature *sensu stricto* is almost beside the point.

## Modelling social release

Critical vulnerability studies received its fullest and most eloquent exposition in *At Risk: Natural Hazards, People’s Vulnerability and Disasters*, again written by Wisner and colleagues. Following the line of inquiry pursued since the 1970s, *At Risk* fleshes out the argument that nature is peripheral to the outcomes and adversity, a fact of life. Whether one can deal with it is a matter of having enough land to farm, adequate access to water, a stash of jewellery or a shed of tools to use in need, and this is strictly ‘determined by social factors’ (Wisner et al. 2004:6). Most fundamental of these are ‘relations of production and flows of surpluses’ (Wisner et al. 2004:91), since they decide what cushions an agent can dispose over. A population is divided into classes, and further into genders, ethnicities, age groups, citizens and migrants with antithetical positions: some wounded on the battlefields of exploitation, discrimination, persecution and oppression, others decked out in shining armour and ready for anything.

**FIGURE 1:**
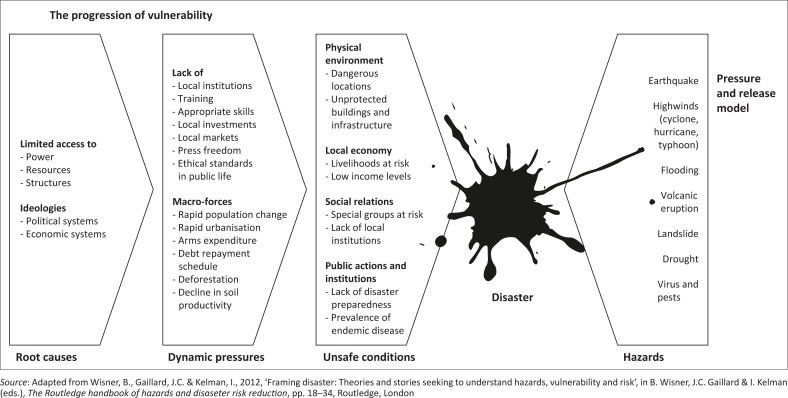
The pressure and release model.

Seeping out from Marxism and into mainstream academia, the basic insight about vulnerability is now easy to come by. Wisner et al. have made it enormously influential through ‘the pressure and release model’ (PAR). Disaster strikes as a clash between two magnitudes: socially determined vulnerability from the one side, natural hazards from the other. In between, people are squeezed or crunched as in a ‘nutcracker’ (Wisner et al. 2004:50). What truly accounts for the result, however, are the goings-on to the left, as depicted in Figure 1. There is a ‘progression of vulnerability’, a sequence of causation running from ‘root causes’ via ‘dynamic pressures’ to ‘unsafe conditions’. Capitalist development marked by deep inequalities (root cause) leads to, among other things, corporate appropriation of land and accelerated urbanisation (dynamic pressures) that impoverish people and drive them to build homes on steep hillsides (unsafe conditions) – and then comes the deluge. The hazard is but a trigger that ‘releases’ the social pressures long accumulated: geophysicalism turned inside out.

But if this is the final word in, or the consummate model for, critical vulnerability studies, a question imposes itself. Where does 21st century climate breakdown fit in?

Barely had the ink dried on the pioneering articles by Wisner and colleagues before it became clear that the scientific community had been overestimating the stability of the climate system and, more precisely, its impermeability to human – that is, social – influence. The belief in the constancy of extreme weather events was put to rest by the discovery of global warming (Weart 2003). Turbulence was injected to the right in the model, the side its originators had wanted to get away from. If the hazards themselves were to redouble in frequency and ferocity and then redouble again, it would be impossible to keep the ‘root causes’ of disaster to the furthest left; for the model to work, the storms and the floods, the landslides and the droughts had to be bracketed as chance events with a fixed probability. Only then could focus be shifted to the perceived social side of the equation.

The analytical difficulties the discovery posed to critical vulnerability studies were apparent in an early attempt to integrate it. Here Wisner and colleagues suggested that climate change is ‘a natural phenomenon but one which is caused by anthropogenic emissions of greenhouse gases’ (O’Brien et al. 2006:68). In the same piece – ironically, in connection with a discussion of Intergovernmental Panel on Climate Change (IPCC) findings – the old article of faith was reiterated: ‘Most disasters, or more correctly, hazards that lead to disasters, cannot be prevented. But their *effects can be mitigated.* (…) Hazards may be natural in origin, but it is the way in which societies have developed that causes them to become disasters’ (O’Brien et al. 2006:65; emphasis added). Such phrasing came close to naturalising climatic hazards and portraying them as acts of God, beyond human influence, impossible to mitigate other than *post festum* – through successful adaptation, in standard terminology.

Similar ad hoc accommodation of climate science is on display in *At Risk*. At one point, Wisner and colleagues classify global warming as yet another ‘dynamic pressure’ to the left, between root causes and unsafe conditions (Wisner et al. 2004:33). This is imprecise. Global warming can scarcely be conceived of as a vector analogous to, say, a debt trap or lack of skills, leading over to unprotected buildings or low incomes. Analytically, it belongs firmly to the right side of the equation, as an *engine of hazards* to which people are more or less vulnerable. And in their most explicit treatment of the subject in their book, Wisner and colleagues recognise precisely this reversal:

In relation to famine, climate change principally acts as a trigger through drought, the shifting of the timing of rainfall and its seasonal patterns (e.g. the Asian monsoon), floods which disrupt the production and distribution of food and the possible spread of disease to humans, livestock and crops. All of these increased risks are almost certainly caused by human action (in relation to greenhouse gases) and relate to social vulnerability and to pre-existing ‘normal’ levels of hazards. But with climate change, human action is responsible for both the generation of people’s vulnerability *and* the increased level of hazard. (Wisner et al. 2004:121; emphasis in original; see also p. 83, 114, 149, 213)

Later on, we learn that ‘consumption of fossil fuels has begun to change the earth’s climate, with a whole series of consequences for food security and health’ (Wisner et al. 2004:195). Such consumption whips up entirely unprecedented amounts of drought, flood, disease and other disastrous occurrences. And that consumption is not, of course, determined by geophysical factors. An extensive body of scholarship has demonstrated that it was initiated and then propagated, accelerated and sustained into this day by capitalist development (e.g. Christophers 2021; Foster, Clark & York 2011; Hanieh 2021; Klein 2014; Malm 2016a, 2016b; Malm & the Zetkin Collective 2021; Wright & Nyberg 2015). If these findings are taken on board, the conceptual framework of critical vulnerability studies starts to fray. Or, more sharply put, the pressure-and-release model explodes in a rightwards direction: the social is no longer on the left side solely. *It has saturated the hazards themselves*.^3^ It turns out that the model rested on an overestimation of the purely natural in nature (or of the purely social in society), tucking away the hazards in a black box when they have themselves become effluents of the prevailing property relations. As critical vulnerability theory once negated geophysicalism, a negation of the negation seems called for.^4^

## Towards a dialectical model of climate disaster

**FIGURE 2:**
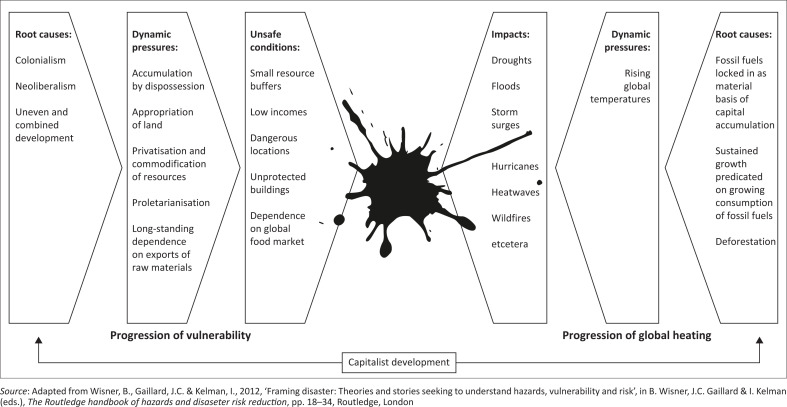
A dialectical model of climatic disaster.

Since models of this sort are meant to be heuristic devices, we can allow ourselves some stylised simplifications. We want a fuller, more dialectical model of climatic disaster. Extending the artwork from Wisner et al., we might picture it something like as shown in Figure 2.

Other factors could be inserted into this model, which is in the nature of a sketch. But the basic point that marks it off from both geophysicalism and pressure-and-release is that *similar social drivers are active on both sides of the equation* – not as omnipotent causes to which nothing can be added, but as non-negligible prime movers. This more fully realises the metaphor of the nutcracker, a tool that works by the same force pressing both handles. The naturalness is removed from natural disasters, in the gestation of the extreme geophysical occurrences *and* the vulnerability of the bodies on which they strike. The enemy that deals the deathblow has first done the damage. A climate disaster lights up the truth about society: about its dependence on fossil fuels *and* its impoverishment of people, which are not necessarily distantly related. A climate disaster releases the pressure of excess energy stored in the system.

Now, if the age of climate breakdown is characterised by uncontrolled speed-up in the production of natural hazards, this would seem to indicate a progressive skewing of the model, with the right side weighing heavier and heavier in disaster causation. Will the splitting of humanity into rich and poor matter when ever-more extreme climate impacts slam into it? Dipesh Chakrabarty has made the (somewhat infamous) argument that this particular crisis suspends the internal divisions of our species: ‘Unlike in the crisis of capitalism, there are no lifeboats here for the rich and privileged’ (Chakrabarty 2009:221). But this holds *only in the very long run*. If average temperature on earth rises by 6 or 8 or 12 degrees Celsius, surely the richest will drown and burn too. Long before that, however, poor people will have perished: during the *early* stages of global heating, which are – and this is of the greatest importance – also the stages when the process can still be slowed down and possibly reversed, the suffering will be concentrated to poles of deprivation (cf. Malm & the Zetkin Collective 2021). If climate breakdown spells the destruction of all manner of biophysical resources, the people who sustain the worst and first losses will, virtually by definition, be those who have the least property rights to such resources and eke out a precarious existence on the margins.

The temporality of global heating is not that of a sudden catastrophic asteroid strike. It is rather like a rising, warming sea that sends off more and more storm surges and category 5 hurricanes, until all walls and levees are – eventually – overtopped. Before that endpoint, the disasters will not cancel out but accentuate the different fates of those with buffers and those without, bringing to the fore the deadly consequences of inequalities, much as in the original framework of critical vulnerability studies. Climate disasters, that is to say, will make ‘the progression of vulnerability’ *more* ubiquitous. The rich and privileged can already now, at an average warming somewhere between 1 and 1.5 degrees, be afflicted – witness the heatwaves in British Columbia, wildfires in California, floods in western Germany in the summer of 2021. Perfect safety from global heating is available for purchase. Aggregate affluence can hide pockets of acute vulnerability (Eriksen et al. 2020). When storm Ida battered New York City in early September, 11 of the 13 casualties lived in basement apartments, drowned in an instant by inrushing water, most of them immigrants and people of colour: the rule in a fractal worldwide pattern of vulnerability (Holpuch 2021). A notion of climate breakdown as a great leveller requires a counter-factual scenario where the left side of the model vanishes, filled instead with exclusively geophysical determinants of vulnerability – without significant inequalities. Such a world does not exist on this planet, and pretending that it does would be to regress to the blindest Cold War-era geophysicalism.

Paradoxically, that paradigm has had a long life in climate science. Modellers have excelled in projecting future impacts on natural systems – the water supply in region X will decrease by Y in response to a temperature increase of Z – while paying scant attention to factors of power, property and class. Research on climate vulnerability has had to play catch-up with the counter-paradigm and incorporate its insights (Adger 2006; Dessai et al. 2004; Eakin & Luers 2006:369; Ford et al. 2010; Leichenko & O’Brien 2006:104–106; O’Brien et al. 2007:85; Pelling 2011:27–28; Ribot et al. 2009:137–138; Thomas et al. 2018:2; Tschakert 2007:381; Turner et al. 2003). Some proponents of the critical school were quick on the draw: in 1994, Michael Watts and colleagues suggested that climate vulnerability is a function of resource endowments, distributed, at the deepest base layer of society, through property relations (Bohle 1994). To take but one example, Egypt is susceptible to harm from global heating, notably sea level rise: but some residents of coastal towns are protected by walls, while others are left in the lurch; some farmers have access to sand, fertilisers, pumps and excavators that allow them to continue farming even as the soil is salinised, while others cannot afford any of this; some entrepreneurs establish fish farms, while artisanal fisherfolk struggle to weather the unpredictable storms, and so on. Most of these differentials can be traced to the specificities of capitalist development in Egypt (Malm 2012; Malm & Esmailian 2012a, 2012b). On the other side of the Atlantic, hurricane Maria in 2017 caused ruin and mass death in Puerto Rico but scratched Texas only lightly: one more occasion for research on climate vulnerability to return to the writings of Wisner and colleagues (Thomas et al. 2018:3–5). They remain indispensable for one half of the problem. What, then, are the merits of a model that adds the other half?

It should be possible to operationalise a dialectical model of climate disaster on several scales of inquiry. One obvious start would be sites where the compound of the two progressions is exceedingly concrete. The Iranian province of Khuzestan is a case in point. To give only the most cursory illustration of the general argument, it is to this scene of contemporary misery we now turn.

## The case of Khuzestan

In July 2021, Khuzestan briefly made headlines outside of Iran, when crowds thronged streets and shouted ‘I am thirsty!’ (e.g. Fassihi 2021). Some upped the ante by chanting slogans against the Islamic Republic and its supreme leader Ayatollah Ali Khamenei. Police and *basij* forces responded with the standard fare of repression, shutting down the Internet and killing at least eight people in what was widely reported as ‘water protests’. The background was straightforward enough: the summer of 2021 was the driest in Iran in at least half a century, precipitation having fallen to 15% of the average in the southwest. As temperatures hovered around 50 degree Celsius, the rivers nourishing Khuzestan – among them Karoun, normally Iran’s longest and sole navigable river – completely dried out downstream, leaving fields baked, water buffaloes dying and potable water in an even shorter supply than usual. On top of this, came another season of dust storms (Karami 2021; Shokri 2021). Popular patience snapped, leading to what might have been parched Iran’s most intense – if short-lived – climate-induced uprising so far.

Khuzestan, incidentally, occupies a special place in the annals of fossil capital. It was here, in the village of Masjed-e Soleyman, that British explorers first struck oil in the Middle East in 1908. The find inaugurated the era of Middle Eastern oil, a pivot for petroleum-fuelled capitalist development in the 20th century (e.g. Mitchell 2011). Khuzestan became a turf of the British Empire, from which it drew oil to its navy in the First World War; as anyone with some knowledge of Middle Eastern history will know, it was to defend this property against the threat of nationalisation that the United Kingdom (UK) and the United States of America (US) engineered the coup against Mohammed Mossadeq in 1953 – an event whose fateful consequences for Iran can hardly be exaggerated (e.g. Keddie 2003; Kinzer 2003). The country was saved for Western capital, for the time being. Abadan remained the single largest oil refinery in the world, the landscape of Khuzestan re-engineered into that of a gas station for foreign customers (Hein & Sedighi 2016; Zandieh, Hekmat & Maghsoudi 2021). In the year after the coup, the colonial construct Anglo-Iranian Oil Company changed its name to British Petroleum, the oil of Khuzestan thereby becoming the main tributary to the entity still known as BP. But the reckoning with the exploitation of Iran had only been postponed. When it exploded in 1979, all oil and gas reserves and installations were nationalised. They have since remained under the tutelage of the National Iranian Oil Company (NIOC), with comparatively limited presence of foreign capital, US corporations entirely absent as the American empire keeps Iran under an increasingly suffocating sanctions regime. Instead, the riches of Khuzestan have come to serve as the foundation of a national bourgeoise sometimes referred to as ‘the millionaire mullahs’ (Malm & Esmailian 2007). The province houses 80% of the oil and 60% of the gas reserves controlled by NIOC.

This history means that the fossil fuels of Khuzestan have made a non-trivial contribution to global heating. In the pathbreaking research conducted by Richard Heede and his colleagues, the companies that have extracted them rank high among the corporate entities most responsible for increasing the atmospheric concentration of CO_2_: for the period 1880–2010, NIOC is on place six, BP on four; in the last four decades of that period, the two carbon majors swapped position (Ekwurzel et al. 2017:585; cf. Climate Accountability Institute 2020; Heede 2014). By 2015, NIOC – that is, for all practical purposes, the enterprise holding Khuzestan – was the third largest corporation in the world, when measured in the greenhouse gas (GHG) emissions its products generated (Griffin 2017:10). This is the capitalist development – what we have elsewhere called the accumulation of ‘primitive fossil capital’ (Malm & the Zetkin Collective 2021) – that forms the burning core of global heating.

And now Khuzestan is itself feeling the heat. Over the past two decades, the heatwaves have become more frequent, severe and long-lasting; in 2017, the provincial capital of Ahvaz hit 54 degrees Celsius, still, as of this writing, the all-time record for temperatures in Asia (al-Jazeera 2021). Evaporation rates have reached extreme levels and increased irrigation demands. Rain-fed fields have had to be abandoned, and in 2018, authorities went so far as to ban rice cultivation (Dehcheshmeh & Ghaedi 2020:8; Khavarian-Garmsir et al. 2019:5; Pakmehr, Yazdanpanah & Baradaran 2020:4–5). Wildfires now regularly tear through the vegetation (e.g. MEHR 2018). The dry soils cannot absorb suddenly arriving water masses, and so the heavy rainfalls in Iran in March 2019 – described as a ‘1-in-100-year event’, the usual euphemism for climate disasters off the charts – brought devastating floods to Khuzestan (National Disaster Management Organization of Iran, United Nations & Presidency Islamic Republic of Iran Plan and Budget Organization 2019). But the new normal is drought, to the extent that one Iranian climatologist has proposed ditching that term for ‘drying out’ – a permanent desiccation of the province (Karami 2021). And then there are the dust storms. They began in 2001 and have since become steadily harsher, winds sweeping up dust from the drying plains and blanketing towns in a greyish-yellowish film that blots out the sun, brings life to a standstill and sends thousands to hospitals with respiratory problems. In the 2010s, Ahwaz rose to become one of world’s most polluted cities, Abadan – the old refinery town – recording 85 days of dust blankets as a new annual average (Khavarian-Garmsir et al. 2019:5; Nada 2021). All of these trends are in line with climate projections for Iran as a whole: it will get even drier and hotter in the decades ahead (e.g. Daneshvar, Ebrahimi & Nejadsoleymani 2019; Hashemi 2015; Rahimi, Malekian & Khalili 2019). Fossil fuels, however, are implicated not only in the production of hazards, but also in vulnerability to the same.

Dust storms can pick up material from far afield, including the similarly dried-out lands of southern Iraq (Javadian, Behrangi & Sorooshian 2019). But they would have been more containable and bearable if Khuzestan had retained its once extensive wetlands. One of the largest, Hour al-Azim, had a water depth of up to 10 m and sprouted an archipelago of lush islets until the turn of the millennium. Then NIOC began to bulldoze its way through the lagoon in search for more oil, draining it, crisscrossing it with roads and platforms and depositing toxic waste into it. The company oversaw the definitive destruction of the Hour al-Azim in the mid-2010s, with assistance from Chinese oil companies flouting the US sanctions (Financial Tribune 2016; Madani 2021:238; Tehran Bureau Correspondent 2015; Zohoorian-Pordel et al. 2017). As a result, an essential geophysical buffer against dust storms – straddling the border with Iraq – was removed and itself turned into a source of dust, enhancing the vulnerability of villages and towns in Khuzestan to the hazard. Such a sequence belongs not to the right side of our dialectical model, but to the left, where, in this case, the dynamic pressure of reckless appropriation of land produces the unsafe conditions of dangerous locations and unprotected buildings. (To make things more (or less) complicated, however, global warming has itself partaken in the drying out of Hour al-Azim.) Gas flaring stacks and other installations in the fossil fuel sector produce heat island effects, further driving up local temperatures (Dehcheshmeh & Ghaedi 2020:12).

This added some political dimensions to fossil-fuelled capitalist development in Iran. Khuzestan is home to the country’s largest Arab population, somewhere between 2 and 5 million, probably still a majority (the southern part of the province used to be called ‘Arabistan’). The dominant class ruling from Tehran is overwhelmingly Persian. Its principal material base is the abundance of fossil fuels, withdrawn from Khuzestan to feed metropolitan accumulation, giving rise to an ethno-political contradiction familiar from other oil-producing nations (notably Saudi Arabia and Nigeria): the centre cannot trust the minority to govern the most precious of resources. Strict military control must be upheld. Arabs have long been suspected of insufficient loyalty to the nation, including sympathies with Saddam Hussein (whose invasion of Iraq laid waste to their land) and Saudi Arabia or separatist aspirations (which occasionally do surface). Conversely, the Arabs of the periphery have long resented the seizure of the riches under their feet. ‘They see the towers of the oil refineries and the flares and all of that money, which is a lot, and it is going out of the province’, a United Nations (UN) envoy observed in the summer of 2005, just after Khuzestan had erupted in the ‘Ahwazi intifada’, in which 130 protestors were killed (Malm & Esmailian 2007:96–7). Some of the most extreme poverty in Iran is found next to those towers.

Unsurprisingly, then, the Arabs of Khuzestan harbour grudges against the central state for sacrificing them to inclement weather. During the floods of 2019, rumours spread about water being redirected from oil installations to Arab villages and fields, ruining the latter on purpose. A video of a distraught Arab man telling the governor that ‘you won’t help us because we are Arab’ went viral, sparking small-scale protests with chants such as ‘Khuzestan has been washed away and [our] leaders have fallen asleep!’ (Centre for Human Rights in Iran 2019; Saidi 2019). In the summer of 2021, the water uprising was triggered by another viral film clip, in which an Arab *sheikh* in traditional dress accused officials of orchestrating the extreme weather: ‘Look, we are not going to leave this land, you brought us floods and drought to make us migrate. We won’t leave, this is our ancestral land’ (Fassihi 2021).

Behind such conspiracy theories is a reality of highly differentiated vulnerability. In Iran, as much as anywhere in the global South, peasants are more sensitive to climatic pressure insofar as they have small farms, rudimentary equipment, few crops to sell, and limited access to credit (Jamshidi et al. 2019; Savari & Zhoolideh 2021). The adaptive measures proposed for Khuzestan’s agriculture – drip irrigation, advanced well pipes, artificial coverings to check evaporation, drought-resistant species, more efficient fertilisers – tend to presuppose precisely the wealth that underdevelopment has denied the peasant masses (Kaabi et al. 2021). ‘New crops need new technology for cultivation and harvesting that we have no access to’, one farmer from the ancient area of Susa recently lamented to a research team (Chenani et al. 2021:6). Clearly, this vulnerability is inversely related to the stream of oil and gas revenues. The same hands that have accumulated capital by producing fossil fuels in Khuzestan have produced vulnerability to the consequences of their combustion *through* that process. The tightness of this dialectic might only be replicated in some other zones of fossil fuel production – neighbouring Iraq and the Niger Delta come to mind – but it could certainly be observed in looser form, still causally relevant, on plenty of other sites and scales.

In Khuzestan, the dialectic has grown so bad that swathes of territory are being depopulated. During the past three decades, more than 1,000 rural settlements have been evacuated primarily because of climatic stress (Dehcheshmeh & Ghaedi 2020:159). Some of the main cities – among them Abadan and Masjed-e Soleyman – are haemorrhaging inhabitants, again primarily because of the heatwaves and the dust storms, global heating having already ‘compromised the human habitability of the region’ (Khavarian-Garmsir et al. 2019:10). Given that these rustic hamlets were turned into boom towns by oil, one is here tempted to see Abdelrahman Munif’s prophecy from 1984 nearing fulfilment:

In twenty or thirty years’ time we shall discover that oil has been a real tragedy for the Arabs, and these giant cities built in the desert will find no one to live in them and their hundreds of thousands of inhabitants will have to begin again their quest for the unknown (…) As a result we shall again have to face a sense of loss and estrangement, this time in complete poverty. (quoted in Nixon 2011:100–101).

But all the migrants fanning out across Iran from Khuzestan are not Arabs, nor are they necessarily abysmally poor. The option of migration can be most affordable for the higher educated and better connected. (Construction and other outdoor workers, on the other hand, are the first to lose their livelihoods when cities shut down because of intolerable heat or dust.) Nor are fossil fuels – in the stage of production or consumption – the sole culprits in Iran’s water crisis. There is a plethora of contributing factors, ranging from aggressive agribusinesses and excessive dam construction to wasteful consumption practices, indirectly related, at most, to oil and gas (Ashraf, Nazemi & AghaKouchak 2021; Madani, AghaKouchak & Mirchi 2016). On the other hand, some of the geopolitical conflicts that have given their share to the wrecking of Khuzestan – the Iran-Iraq War, the Gulf War, the American sanctions – are rather closely related to the struggle for the black gold (e.g. Madani 2021). Whatever its exact role in these events, one thing is not in doubt: the Iranian branch of fossil capital is not foregoing sources of profit. In November 2019, then-president Hassan Rouhani announced the discovery of a new giant oilfield in Khuzestan, adding one third to the nation’s reserves in one stroke – ‘a small gift by the government to the people of Iran’, he called it (Altaher & Robinson 2019). In January 2021, the largest gas refinery in the Middle East went online, slated to produce 56m cubic metres of processed gas per day and some $700m in profit per year, in the eastern corner of the province, in a rural area abutting the Gulf (Tehran Times 2021). And this is unlikely to have been the last acts of business-as-usual in Khuzestan.

## Shifting revolution to the right

The political gist of critical vulnerability studies was never very hard to spot. ‘Only radical changes in the organisation of production and in access to political power will affect in a large number of direct and indirect ways vulnerability to disaster’, wrote Ben Wisner in the journal *Disasters* in 1979 (p. 305). In *Interpretations of Calamity*, he and his colleagues proclaimed that ‘the only way to reduce vulnerability is to concentrate disaster planning within development planning, and that development planning context must be, broadly speaking, socialist’ (Susman et al. 1983:280). The authors of *At Risk* take notice of a critic who charges them with views that ‘simply call for overall social revolution’ (Keith Smith quoted in Wisner et al. 2004:27), and they do not bother to refute the claim. Instead, they reiterate that the best recipe for protection against disaster is an assault on the ‘root causes’ of vulnerability through a ‘revolution or major realignment in the balance of class forces’ (Wisner et al. 2004:91). All of this would happen on the left side of their model.

When critical vulnerability studies were first applied to the problem of climate change, they pointed to a rather sanguine policy recommendation: the right dose of social transformation can bring the problem under control. If ‘it is not so much the droughts or floods that are alarming, but people’s vulnerability to the consequences associated with them’ (Ribot et al. [1996] 2009:133), then eradication of such vulnerability would silence the alarm. The importance of globalised markets and state systems in determining vulnerability, one researcher argued, ‘indicates that climate change, while a significant challenge, *can be managed* with the correct adaptive responses’ (Ford et al. 2010:383; emphasis added). While ‘responses’ have another inflection than ‘revolution’, the essential point is the same: make people equal, heal the wounds sustained on the battlefield and the sword will bounce. Today, such a position appears outdated, or, if you will, half-revolutionary.

Wisner recently called for the phrase ‘disaster risk reduction’ to be replaced with the slogan ‘resist disaster risk *creation*’ (Wisner 2017:3, emphasis in original). His immediate reference was to megaprojects – including oil infrastructure burying coastal wetlands in the southern US – the main result of which is deepened vulnerability. But that too was a prescription to the left side. In the same vein, the water protests of Khuzestan have voiced bitterness against the Islamic Republic for leaving people to their fate in times of flood and drought. But they have yet to target the platforms and the refineries. Until that happens – until revolutionary struggle breaks out on the right side – we are, it seems, doomed to facing an ever-rising tide of disasters. Why is this turn almost nowhere to be seen? The answers to that question are beyond the scope of this essay. They might fill whole libraries of their own.

## Footnotes

1 At a closer look, this is a fallacy: it does not follow from an observation of climate stability ‘in recent years’ that ‘the probability of the extreme physical occurrence is constant’ – constancy is not proven by conditions over a few years. If climate was stable in the 1970s, it could well collapse in the 2010s. A statement like this should perhaps be read as an indication of the perception of the climate system as having a guaranteed stability, still widespread in the 1970s: see Weart (2003).

2 It has been argued that Hewitt (1983a) and his contributors ‘simply reinvented the wheel’, since critical voices already in the 19th century claimed that famine in the colonies were the product not of nature, but of colonial oppression (Brookfield 1999:3). Similar claims were, of course, made by Karl Marx, for example in the case of the Irish potato famine (Marx 1990 [1867]). But this was apparently a wheel in need of reinvention – with Marx’s writings as manuals – given its enormous subsequent influence in vulnerability research.

3 This is emphatically not to suggest that nature is hereby ‘constructed’ or ‘produced’ or ‘ended’ by society: rather to the contrary. For some considerations on this dialectic, see Malm (2018).

4 In fact, Wisner and two colleagues did attempt to take account of the social saturation of left-and-right hand sides in a mechanical manner by adding a feedback arrow to PAR. However, this amounts only to an acknowledgement of the problem posed for PAR, not a detailed solution. See Wisner, Gaillard & Kelman (2012).
